# Family Check-Up Online: Effects of a Virtual Randomized Trial on Parent Stress, Parenting, and Child Outcomes in Early Adolescence

**DOI:** 10.1007/s11121-024-01725-3

**Published:** 2024-09-24

**Authors:** Anne Marie Mauricio, Katherine A. Hails, Allison S. Caruthers, Arin M. Connell, Elizabeth A. Stormshak

**Affiliations:** 1 Prevention Science Institute, 6217 University of Oregon, Eugene, OR 97403 USA; 2 Department of Psychological Sciences, Case Western Reserve University, Cleveland, OH 44106 USA

**Keywords:** Youth outcomes, Parenting, COVID-19 pandemic, EHealth

## Abstract

We adapted the Family Check-Up Online (FCU-O) (1) to support families coping with pandemic-related stressors to prevent behavioral and emotional problems among middle school youth and (2) for smartphone delivery to increase access and reach during the COVID-19 pandemic. This study evaluated the direct and indirect effects of the adapted FCU-O at 4-months post-baseline. The FCU-O combines online parenting support with telephone coaching. Participants were primary caregivers of children ages 10 to 14 years. Eligibility included endorsing depression on the PHQ-2 or significant stress on a 4-item version of the Perceived Stress Scale. We randomly assigned participants to the adapted FCU-O (*N* = 74) or a waitlist control condition (*N* = 87). Participants predominantly self-identified as female (95%), 42.77 years old on average, and White (84.6%). Outcomes included caregiver reports of perceived stress and parenting, and youth conduct problems and depressive symptoms. Using a multilevel modeling approach, we tested intent-to-treat intervention effects at 4-months, with time points nested within participants. The FCU-O reduced caregiver stress and improved proactive parenting and limit setting but had no effects on youth outcomes. Effect sizes were small to moderate (Cohen’s *d* ranged from .37 to .57). We examined indirect effects on youth outcomes at 4-months via changes in caregiver stress and parenting at 2-months. Mediation analyses suggested indirect effects on youth depressive symptoms via reductions in caregiver stress and increases in proactive parenting. Results indicate the FCU-O has potential as a public health intervention for families facing extreme stressors such as those during the COVID-19 pandemic. ClinicalTrials.gov Identifier: NCT 05117099.

**Supplementary Information:**

The online version contains supplementary material available at 10.1007/s11121-024-01725-3.

## Introduction

Youth with mental, emotional, and behavioral health problems have been underserved for decades, with only one in five youth receiving the services they need, and 62% of youth with mental health distress and histories of adversity receiving no services (Finkelhor et al., 2021). Lack of service provision, along with rates of mental health problems, substantially increased for at-risk youth during the coronavirus 2019 (COVID-19) pandemic (Singh et al., 2020). New research suggests 25% of youth report depression in the clinical range—double the rates prior to the pandemic (Racine et al., 2021). The effects of the pandemic were most evident among socially and economically marginalized youth and families and exaggerated existing health and academic-related disparities (Thomeer et al., 2023). Most youth mental health interventions include individual in-person treatments for children and families. These treatments are efficacious (Sandler et al., 2014), but there are limited mental health providers available to deliver this level of support. According to a recent survey by the American Psychological Association, 80% of providers reported increased demand and growing waitlists for services and an inability to meet the demand in their communities (American Psychological Association., 2021). Moreover, the mental health provider workforce across different healthcare settings has declined since COVID-19, and shortages in behavioral health outpatient care are not planned to recover to pre-pandemic levels (Eisenberg et al., 2022). Families of color have been found to have the most difficulty accessing mental health services (Thomeer et al., 2023).

Telehealth models, which reduce in-person treatment barriers such as transportation, childcare, and scheduling, can increase intervention access and reach (Butzner & Cuffee, 2021). In addition to increased flexibility with respect to time and schedule of participation, telehealth and online modalities normalize help-seeking and reduce associated stigma (Metzler et al., 2012), particularly in the current digital context in which engaging in self-help mobile applications is increasingly normative. The recent pandemic highlighted the need for digital interventions, accelerating the development of such interventions, many of which have shown encouraging results across multiple outcomes (Eyllon et al., 2022). The difficulty of accessing mental health services, particularly since the pandemic and for families of color, highlights the potential of online interventions to support underserved families during times of crisis.

### The Family Check-Up Online Intervention

The Family Check-Up (FCU) is a brief, strength-based intervention tailored to a family’s needs and goals that applies motivational interviewing strategies. The FCU intervention model is grounded in research and theory supporting changes in parenting and family processes that link to improved youth outcomes, including internalizing and externalizing behaviors (Rudo-Stern et al., 2019). Across multiple randomized trials with ethnically and socioeconomically diverse families, the FCU demonstrated effects on multiple youth outcomes, including depression (Connell & Dishion, 2008) and conduct problems as well as antisocial behavior (Shelleby et al., 2018; Fosco et al., 2014). These effects were mediated by changes in parenting (Fosco et al., 2012), family processes such as family conflict (Fosco et al., 2014), and caregiver depression (Reuben et al., 2015).

The original FCU model was delivered in-person and begins with an initial interview to develop rapport and motivate caregiver engagement. After the interview, families complete an assessment with questionnaires and videotaped observational caregiver-child interaction tasks. Following the assessment is a feedback session, where interventionists share assessment findings and collaborate with caregivers to identify intervention goals and tailor follow-up services to meet goals. Follow-up services include content from the Everyday Parenting curriculum (Dishion et al., 2011). Unlike most parenting curricula, for which optimal dosage is the full curriculum, Everyday Parenting is modular, and delivery is customized for each family.

The in-person FCU was translated to a telehealth model, FCU-Online (FCU-O), which includes an asynchronous online program delivered with supplemental telephone coaching. The original FCU-O, available via computer only, was developed with feedback from schools and providers in response to limited staffing and provider availability in schools and community settings that challenged the feasibility of the in-person model (Danaher et al., 2018). It includes an assessment, computer-generated feedback, and intervention modules with content from the Everyday Parenting curriculum (Dishion et al., 2011), including positive parenting, rules and consequences, support for school success, and communication. The FCU-O applies empirically supported eHealth strategies, such as videos and interactive activities with synchronized text message reminders to encourage program engagement (Lynch & Horton, 2016). The original FCU-O was tested in an efficacy trial with families of middle school students (Stormshak et al., 2019). Results demonstrated intervention effects on parenting self-efficacy, a key mechanism of change, and on children’s emotional and behavioral problems at 3-months post-baseline in comparison to a waitlist control. Engagement was high, with 73% of caregivers participating in the FCU-O, supporting the acceptability and feasibility of the FCU-O.

The original FCU-O was adapted in response to the COVID-19 pandemic, which impacted families in many ways, including economic stress and reductions in mental health, academic functioning, and health behaviors such as sleep (Bates et al., 2021; Glynn et al., 2021). These effects were pronounced for children struggling academically or with mental health and behavioral problems before the pandemic (Bambra et al., 2020). The adapted FCU-O was designed to support families as they navigated pandemic-related stressors such as online schooling, isolation, and mental health problems. Like the original FCU-O, the FCU-O adapted for the pandemic combines online parenting support with telephone coaching to promote the practice of skills learned using the online program. However, while the original FCU-O was only available via computer, the adapted FCU-O is accessible via smartphone to increase accessibility and reach. Moreover, the modules and corresponding assessments were significantly shortened to enhance feasibility, and more interactive practices were included to support caregiver uptake of skills.

### The Current Study

This study is an evaluation of the FCU-O COVID-19 adaptation. A previous study (Connell & Stormshak, 2023) demonstrated intervention effects for the FCU-O COVID-19 adaptation 2-months post-baseline on caregiver stress and parenting. The current study builds on this previous study by examining effects on caregiver stress and parenting at 4-months post-baseline and by examining intervention effects on child outcomes. We hypothesized that FCU-O would decrease caregiver stress and improve parenting, specifically limit setting, negative parenting, and proactive parenting, as well as decrease child depressive symptoms and conduct problem behaviors. We also examined whether changes in stress and parenting at 2-months post-baseline mediated FCU-O effects on child outcomes. While FCU research has established parenting skills as an underlying mechanism of change (Rudo-Stern et al., 2019), this is the first FCU study to specifically examine caregiver stress as a mediating mechanism of change for child outcomes. However, parenting stress has been linked to long-term child mental health and has been a target among parenting interventions aimed at improving child mental health (Moreland et al., 2016). In the current study, pandemic-related caregiver stress was exceptionally high (Adams et al., 2021) and may have been particularly salient as a risk factor for child outcomes; as such, we hypothesized that decreasing stress would indirectly decrease youth depressive symptoms and conduct problem behaviors.

## Methods

### Procedures

Families were recruited between December 2021 and March 2022. Initially, our team attempted our typical recruitment approach that involves direct collaboration with schools to share information about the study via flyers, newsletters, and verbally. However, given post-pandemic demands that challenged schools’ daily operations, schools had limited resources to support recruitment efforts, and we had limited success recruiting families. Moreover, starting in January 2022, districts across Oregon had intermittent pauses to in-person schooling for weeks at a time due to increasing rates of COVID-19, challenging direct recruitment through schools. As such, in January 2022, we initiated a collaboration with the University of Oregon (UO) Monitoring and Assessment Program (MAP) to support recruitment. As a public health response to the pandemic, UO launched a COVID-19 testing lab, MAP, that operated from 2020 to 2023 (https://research.uoregon.edu/covid-19-map). MAP collaborated with 26 Oregon school districts and 168 schools to disseminate COVID-19 testing kits to families. Families interested in the testing program could register to participate. We pivoted from relying on direct recruitment through schools to invite study participation by sending flyers about the study in the COVID-19 testing kits distributed to families by schools. At the start of each quarter, MAP sent testing kits including our flyer to the school for students registered in the testing program. Students took the kits home, or caregivers retrieved them from the school. The kits contained individually wrapped tests for collecting weekly saliva samples for testing, which students returned to schools weekly.

**Fig. 1:**
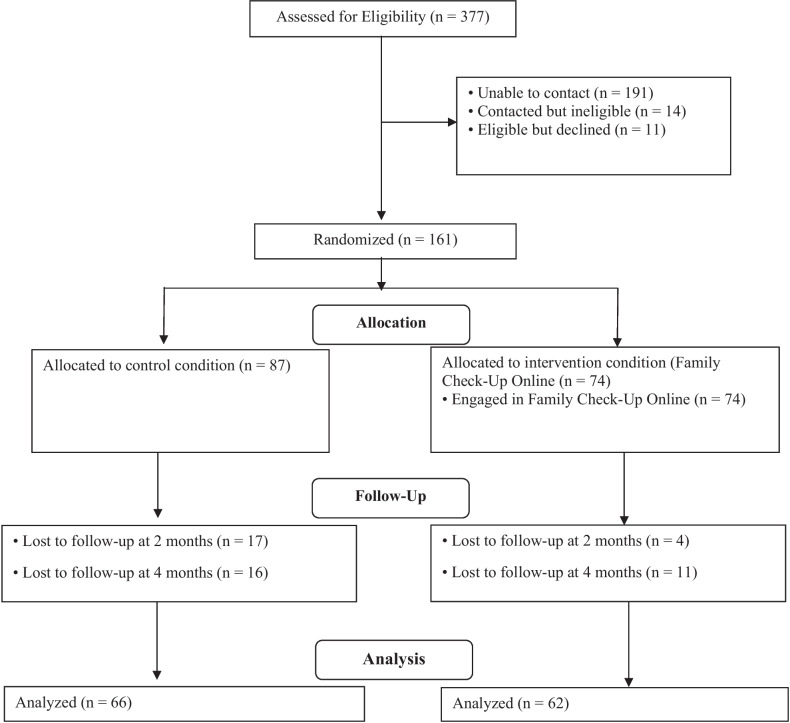
CONSORT flowchart of participants

The flyer provided study information and directed caregivers to the study website. Interested caregivers contacted the study team via the website or a phone call, and a research team member offered more information, confirmed eligibility, and reviewed informed consent for their and their adolescent’s participation. Next, the research team member e-mailed eligible caregivers a digital copy of the informed consent to sign. Then, caregivers completed the baseline assessment via an online or telephone survey per participant’s choice, after which the research team member randomized them to the intervention or waitlist condition. Families were block-randomized by child gender using a computer-generated random sequence. Caregivers in both conditions completed baseline, 2-month, and 4-month follow-up assessments. Across all waves, 97% completed the interviews online. Caregivers received $75 for each assessment. Researchers were not blind to condition (see Fig. 1 for CONSORT diagram).

### Participants

Participants were primary caregivers (*N* = 161; 93% biological parent, 5% adopted parent, 2% other legal guardian) of children aged 10 to 14 years. To be eligible, caregivers had to endorse depression on the Patient Health Questionnaire-2 (PHQ-2; Lowe et al., 2005) or significant stress on a 4-item version of the Perceived Stress Scale (PSS; Cohen et al., 1994) and have access to e-mail and a smartphone. Caregivers were predominantly female (*n* = 153; 95%), 42.77 years old on average (*SD* = 6.65), and married/cohabiting (*n* = 134; 83.2%). Caregivers reported whether they self-identify as Latine; 20.4% of caregivers self-identified as Latine. Caregivers also self-reported on their race; 84.6% self-identified as White, 11.7% as multiracial, 1.9% as Asian, 0.6% as African American, and 1.2% did not answer. Most caregivers (66.7%) reported at least a 4-year college degree. The median family income was between $80,000 and $89,000, but 14.9% (*n* = 24) had annual incomes below poverty. Youth were balanced by gender (50.9% female), and the average age was 12. See Supplemental Table 1 for demographic data by intervention assignment. There were no significant differences in demographic factors across conditions. Our data represented 75 schools and 23 districts. Across districts, students were 20.9% Latine, 64.2% White, 7.1% multiracial, 3.3% Asian, 2.1% African American, and 2.4% other. Fifteen percent of students were participating in remote learning, and 51% were eligible for free or reduced lunch (https://www.ode.state.or.us/data/ReportCard/Reports/InstList).

### FCU-O Intervention Protocol

The FCU-O COVID-19 adaptation evaluated in this study was available in English and Spanish, with corresponding telephone coaching. The proposed coaching model is an initial session to motivate caregiver engagement and collaboratively identify a parenting goal (e.g., more effectively set rules), with approximately five follow-up sessions corresponding with engagement in each of the five FCU-O modules. Follow-up sessions support and individualize caregivers’ practice of skills learned in each module. Sessions are approximately 20 min. The coaching model is tailored to caregivers’ needs and wants, with some caregivers only engaging in an initial session and some caregivers engaging in more than one session per module. The length of coaching sessions also varies. At all contacts, coaches employ motivational interviewing strategies to engage families in the change process, facilitate goal setting, and support families in behavioral skill development.

For the FCU-O COVID-19 adaptation, we created a new module “Healthy Behaviors for Stressful Times,” focused on supporting families to create healthy behavioral routines and structure for reducing anxiety and depression during the pandemic. Development of the new module included content from the *Everyday Parenting* (Dishion et al., 2011) and was guided by evidence-based parenting practices to promote healthy coping and well-being during stressful times, such as physical exercise, family and social support activities, managing screen time, structured morning, evening, and meal routines, and academic support (Bates et al., 2021; Glynn et al., 2021). We developed content for the new module in the year prior to the start of the current study using a participatory process similar to the one outlined in Danaher et al. (2018) and Stormshak et al. (2019) that relied on caregiver input related to acceptability of the new content. We piloted the new module with a small sample of middle school families immediately prior to the start of this study. Supplemental Figure 1 presents sample FCU-O content and activities.

Coaches were licensed, masters-level clinicians trained in the FCU-O. After training, audio recordings of coaches’ first two sessions were assessed for fidelity by coaches and their trainer-supervisor using the empirically validated COACH FCU fidelity rating system (Smith et al., 2013). For ongoing fidelity monitoring during the trial, coaches participated in group supervision twice monthly during which they and their trainer-supervisor listened to audio recordings of sessions and collectively assessed fidelity using the COACH. The trainer-supervisor was a long-term clinician on the research team who was certified as a FCU trainer-supervisor. Coaches were randomly assigned to intervention families following the baseline assessment, after which they contacted families for an initial session to support use of the FCU-O, set goals, and provide a framework for coaching. Two coaches served most families (coach 1 = 32 families, coach 2 = 40 families), and a third coach worked with the two remaining families.

### Waitlist Control Condition

During the trial, the waitlist control condition completed assessments on schedule. After the trial, they were offered the same FCU-O content as the intervention group and a slightly varied coaching model. Similar to the intervention condition, caregivers were offered an initial session to facilitate the use of the FCU-O and set goals, during which coaches also invited caregivers to contact them for additional sessions as needed. While sessions after the initial session were caregiver initiated, there were no limits on the number of coaching sessions.

### Measures

#### Demographic Characteristics and COVID-19 Effects

Caregivers completed demographic questions for family members, including gender, age, race/ethnicity, and indices of Socioeconomic Status (SES). They also reported on challenges experienced during the pandemic (e.g., job loss and experiences with remote learning). A risk index related to SES was created based on six dichotomous indicators: high school diploma or GED, status is single/unpartnered, income below poverty threshold (relative to family size), recipient of financial assistance (e.g., food stamps), currently unemployed, and family home is overcrowded (bottom one-third of the sample for room-to-occupant ratio).

#### Caregiver Perceived Stress

Caregivers completed the 14-item Perceived Stress Scale (PSS; Cohen et al., 1994). The PSS items are scaled from 0 (*never*) to 4 (*fairly often*), with higher scores reflecting greater past-month stress (baseline alpha = 0.90). For screening purposes, a 4-item version of the PSS was used, with scores of 2 or above on any item (sometimes) used to define eligibility.

#### Caregiver Depressive Symptom Screener

We used the self-report 2-item Patient Health Questionnaire (PHQ-2; Kroenke et al., 2001) as a depression screener to assess caregiver eligibility. Caregivers reported on the frequency they experienced symptoms of depressed mood in the past 2 weeks; responses range from 0 (*not at all*) to 3 (*nearly every day*)*.* Eligibility included scores 1 or above (experiencing symptoms for several days), a cutoff with high sensitivity for screening.

#### Parenting Skills

Caregivers completed three subscales of the Parenting Young Children scale adapted for adolescents (PARYC; McEachern et al., 2012): (1) proactive parenting, 7-items reflecting planning ahead to minimize problems with youth (e.g., breaking tasks into small steps; baseline alpha = 0.79); (2) limit setting, 7-items reflecting setting rules, expectations, and consequences (baseline alpha = 0.76); and (3) negative/coercive parenting, 7-items reflecting parental coercion and criticism of youth (baseline alpha = 0.70). For all subscales, the response scale ranges from 0 (*never*) to 4 (*very often*), with higher scores reflecting greater levels of the parenting behavior.

#### Youth Conduct Problem Behaviors and Depressive Symptoms

Caregivers completed the 5-item conduct problems subscale of the Strengths and Difficulties Questionnaire (SDQ; Goodman, 1997; baseline alpha = 0.74) with items on a 3-point scale (*0* = *not true to 2* = *certainly true*). They completed the Patient Health Questionnaire-9 to report on child depression (PHQ-9; Kroenke et al., 2001; baseline alpha = 0.85). Caregiver reports on child problem behaviors and depressive symptoms were based on the 2 weeks prior to the interview.

### Data Analyses

Descriptive analyses examined COVID-19 impacts (e.g., job changes), FCU-O engagement, and factors associated with attrition at the 4-month assessment. Correlational analyses examined associations between demographics and baseline levels of treatment targets to identify covariates for intervention-outcome analyses. We tested intervention effects using a multilevel modeling approach, with time points nested within participants, and an unstructured covariance matrix. An advantage of multilevel modeling is it supports the inclusion of all available time points of data. Analyses included time (baseline, 2- and 4-months), a binary treatment-group variable (0 = waitlist control; 1 = FCU-O), and a treatment × time interaction. We conducted separate analyses for each outcome and controlled for covariates identified as relevant in correlational analyses. When multilevel model results showed a significant time × treatment interaction, we conducted follow-up contrasts to compare intervention and control groups at each assessment employing Bonferroni adjustments for multiple comparisons. A priori power analyses indicated 80% power for a Minimum Detectable Effect of Cohen’s *d* ≥ 0.24 (a small to moderate effect). Relevant tests of fixed effects from the multilevel analyses are reported in Table 1; estimated betas for model parameters are shown in Supplemental Table 2.

**Table 1:** Tests of fixed effects from the multilevel treatment outcome analyses

Outcome variable	Time main effect	Treatment main effect	Time × treatment effect	Baseline assessment, *M* (SE), Cohen’s *d*	Baseline assessment, *M* (SE), Cohen’s *d*	Baseline assessment, *M* (SE), Cohen’s *d*
Perceived stress	*F*(2, 141.15) = 13.32, *p* < .001	*F*(1, 159.15) = 4.14; *p* = .04	*F*(2, 141.16) = 7.91, *p* < .001	Control: 1.82 (.06) FCU-O: 1.83 (.06) *ns*	Control: 1.80 (.06) FCU-O: 1.53 (.06) *d* = .50	Control: 1.75 (.06) FCU-O: 1.54 (.07) *d* = .37
Negative parenting	*F*(2, 139.40) = 28.96, *p* < .001	*F*(1, 151.86) = 0.53, *ns*	*F*(2, 139.42) = 13.10, *p* < .001	Control: 1.16 (.06) FCU-O: 1.31 (.07) *ns*	Control: 1.13 (.06) FCU-O: 0.96 (.06) *d* = .30	Control: 1.04 (.06) FCU-O: 0.88 (.06) *d* = .29
Proactive parenting	*F*(2, 141.92) = 0.84, *ns*	*F*(1, 156.13) = 8.81, *p* = .003	*F*(2, 141.94) = 2.29, *p* = .10	Control: 2.69 (.07) FCU-O: 2.82 (.07) *ns*	Control: 2.66 (.06) FCU-O: 2.95 (.07) *d* = .46	Control: 2.65 (.07) FCU-O: 2.96 (.07) *d* = .51
Parental limit setting	*F*(2, 141.59) = 1.73, *ns*	*F*(1, 152.61) = 6.81, *p* = .01	*F*(2, 141.60) = 1.36, *ns*	Control: 2.76 (.06) FCU-O: 2.85 (.06) *ns*	Control: 2.78 (.06) FCU-O: 3.00 (.06) *d* = .41	Control: 2.76 (.06) FCU-O: 2.97 (.06) *d* = .40
Youth conduct problems	*F*(2, 139.72) = 6.56, *p* = .002	*F*(1, 152.63) = 0.05, *ns*	*F*(2, 139.72) = 1.06, *ns*			
Youth depression	*F*(2, 140.72) = 10.34, *p* < .001	*F*(1, 153.46) = 0.46, *ns*	*F*(2, 140.73) = 0.65, *ns*			

*FCU-O* Family Check-Up Online

We tested indirect effects of the FCU-O on youth outcomes at 4-months via changes in caregiver-level variables (perceived stress, proactive parenting, limit setting, negative parenting) at 2-months to preserve the temporal ordering of caregiver and youth outcomes. We controlled for baseline levels of caregiver and youth variables, so intervention effects reflect treatment impact on changes in the respective variables from baseline. See Supplemental Fig. 2. We conducted four analyses in Mplus 8.7 (Muthén and Muthén, 2022), examining the indirect effects of each caregiver-level outcome on youth depression and conduct problems. Statistical tests of indirect effects were estimated using the delta method (MacKinnon et al., 2002).

## Results

### Preliminary Analyses

#### COVID-19 Impacts

By the start of this study (December 2021), most youth were receiving in-person instruction (*n* = 140; 87.5%). Oregon implemented remote learning on March 19, 2020, and returned to hybrid or fully in-person instruction by April 19, 2021. During remote instruction, youth spent 3–4 h per day on remote learning. Caregivers spent 1–2 h per day assisting with remote instruction, with most (*n* = 110; 68.8%) reporting remote instruction caused “a lot” of stress in their family (a score of 4 on a scale from 1 = “none” to 4 = “a lot”). Additional COVID-related stressors included having an immediate family member diagnosed with COVID-19 (44.7%), pandemic-driven transitions to remote work (42%), job loss (21.6%), or reductions in work hours (26.5%). There were no significant differences between treatment and waitlist control groups in any COVID-related stressors.

#### Attrition Analysis

One hundred forty-three caregivers (88.8%) completed 2-month follow-up assessments, and 135 (83.9%) completed 4-month follow-up assessments. Retention at the 4-month assessment was not related to intervention assignment (*χ*^2^[1] = 0.32, *ns*), caregiver gender (*χ*^2^[1] = 0.21, *ns*), youth gender (*χ*^2^[1] = 3.62, *ns*), marital/cohabiting status (*χ*^2^[1] = 0.86, *ns*), caregiver race (*χ*^2^[4] = 3.23, *ns*), or caregiver Latine ethnicity (*χ*^2^[1] = 3.56, *ns*). Similarly, missing data at 4-months was unrelated to baseline levels of outcome variables, including perceived stress (*F*(1, 157) = 0.02, *ns*), negative parenting (*F*(1, 158) = 2.38, *ns*), limit setting (*F*(1, 158) = 0.01, *ns*), proactive parenting (*F*(1, 159) = 0.26, *ns*), youth conduct problems (*F*(1, 159) = 1.55, *ns*), or youth depressive symptoms (*F*(1, 159) = 2.22, *ns*).

#### FCU-O Engagement

Caregivers in the intervention condition spent an average of 137.05 min reviewing online content (*SD* = 54.49, range = 15.61 to 352.86), visited the app 10.37 times on average (*SD* = 4.34, range = 1 to 25), completed an average of 5.45 coaching sessions (*SD* = 1.73, range = 1 to 11), and spent an average of 160.30 min in coaching (*SD* = 84.25, range = 0 to 472); coaching sessions were 29.37 min on average (*SD* = 13.29, range = 0 to 71). Caregivers who engaged with online content were more likely to engage in coaching, and caregivers with a higher SES risk index spent more time engaged in the FCU-O (see Supplemental Table 3).

#### Baseline Correlations

We present associations between demographic factors and caregiver and youth functioning at baseline in Supplemental Table 4. Youth age was negatively related to caregiver reports of proactive parenting. Youth gender was unrelated to caregiver or youth functioning. SES risk correlated positively with racial/ethnic minority status (White = 0, racial/ethnic minority = 1) and with perceived stress, engagement in negative and proactive parenting behaviors, and youth conduct problems and depressive symptoms. Given these results, youth age, racial/ ethnic minority status, and SES risk were included as covariates in treatment outcome analyses.

### Treatment Outcome Analyses

Multilevel model results for perceived stress showed a significant main effect for time and treatment and a significant time × treatment interaction. Follow-up contrasts showed significant differences between intervention and control groups at the 2-month and 4-month follow-up but not at baseline. There was a significant main effect of SES-related risks *F*(1, 159.93) = 10.88, *p* < 0.001, with more SES risk associated with greater stress.

Multilevel model results for negative parenting showed a significant main effect for time, but the main effect for treatment was not significant. The time × treatment interaction was significant, and follow-up contrasts showed a trend-level difference between intervention and control at the 2-month and 4-month follow-up assessments but not at baseline. There was a significant main effect of SES risk, *F*(1, 151.32) = 14.83, *p* < 0.001, with more risk associated with more negative parenting.

Multilevel model results for proactive parenting showed a nonsignificant main effect for time, a significant main effect for treatment, and a trend-level time × treatment interaction. Although the treatment × time interaction was only trend-level, follow-up analyses compared intervention and control groups at each time point for consistency. Intervention and control groups were not significantly different at baseline but were significantly different at the 2-month and 4-month assessments. There was a significant main effect of youth age, *F*(1, 157.43) = 9.39, *p* = 0.003, with caregivers of older youth reporting less engagement in proactive parenting.

Multilevel model results for limit setting showed a nonsignificant main effect for time, a significant main effect for treatment, and nonsignificant time × treatment interaction. Although only the treatment main effect was significant, follow-up analyses examined time-specific differences between treatment and control groups to mirror follow-up analyses for other outcomes. Intervention and control groups were not significantly different at baseline but were significantly different at the 2-month and 4-month follow-up assessments. There were no significant covariate effects.

For youth conduct problems and depression, multilevel model results showed a significant main effect for time but main effects for treatment and the time × treatment interaction effects were not significant. There was a significant main effect of SES-related risk for conduct problems (*F*(1, 151.40) = 11.32, *p* < 0.001) and depression (*F*(1, 153.86) = 11.03, *p* < 0.01), with elevated risk associated with greater conduct problems and depression in youth.

### Indirect Effects of Intervention on Youth Outcomes

The indirect effects model for caregivers’ perceived stress provided reasonable fit to the data (*χ*^2^ = 18.65, *df* = 12, *p* = 0.10, CFI = 0.98, TLI = 0.96, RMSEA = 0.06, SRMR = 0.04). Controlling for baseline stress, the FCU-O significantly predicted reductions in stress at 2-months (estimate =  − 0.28, *SE* = 0.07, *p* < 0.001). In turn, greater stress at 2-months significantly predicted elevated youth depressive symptoms at 4-months (estimate = 0.14, *SE* = 0.06, *p* = 0.02), controlling for baseline levels of youth depression (estimate = 0.71, *SE* = 0.05, *p* < 0.001). Although the FCU-O had no direct effects on youth depression (estimate = 0.07, *SE* = 0.14), the indirect effect of FCU-O on youth depression via changes in stress was significant (estimate =  − 0.04, *SE* = 0.02, *p* = 0.04). Youth conduct problems at 4-months were not significantly related to the intervention (estimate = 0.04, *SE* = 0.20) or to stress at 2-months (estimate = 0.09, *SE* = 0.06, *p* = 0.09), and the indirect effect of treatment on youth conduct problems via changes in stress was not significant (estimate =  − 0.09, *SE* = 0.05).

The indirect effects model for negative parenting provided reasonable fit to the data (*χ*^2^ = 30.97, *df* = 18, *p* = 0.03, CFI = 0.97, TLI = 0.94, RMSEA = 0.06, SRMR = 0.05). Controlling for baseline negative parenting, the FCU-O significantly predicted negative parenting at 2-months (estimate =  − 0.26, *SE* = 0.06, *p* < 0.001). However, negative parenting at 2-months was unrelated to youth depression (estimate = 0.05, *SE* = 0.06) or to youth conduct problems at the 4-month follow-up assessment (estimate = 0.20, *SE* = 0.22). The indirect effects of treatment on youth depression (estimate =  − 0.01, *SE* = 0.02) and conduct problems (estimate =  − 0.01, *SE* = 0.02) via negative parenting were not significant.

The indirect effects model for proactive parenting provided strong fit to the data (*χ*^2^ = 2.99, *df* = 6, *p* = 0.81, CFI = 1.00, TLI = 1.00, RMSEA = 0.02, SRMR = 0.00). The FCU-O predicted increases in proactive parenting at 2-months (estimate = 0.21, SE = 0.08, *p* = 0.01), controlling for baseline proactive parenting. Controlling for baseline depression, youth depression at 4-months was not related to treatment status (estimate = 0.04, *SE* = 0.07) but was significantly related to proactive parenting at 2-months (estimate =  − 0.14, *SE* = 0.05, *p* = 0.009). The indirect effect of treatment on youth depression at 4-months via improvements in proactive parenting at 2-months was observed at a statistical trend-level (estimate =  − 0.03, *SE* = 0.02, *p* = 0.07). Youth conduct problems at 4-months were related to conduct problems at baseline (estimate = 0.80, *SE* = 0.03, *p* < 0.001), but not to treatment assignment (estimate = 0.02, *SE* = 0.05) or proactive parenting at 2-months (estimate =  − 0.09, SE = 0.06), and the indirect effect of the intervention on conduct problems via proactive parenting was not significant (estimate =  − 0.01, *SE* = 0.01).

The indirect effects model for limit setting provided good fit to the data (*χ*^2^ = 4.68, *df* = 6, *p* = 0.59, CFI = 1.00, TLI = 1.00, RMSEA = 0.00, SRMR = 0.03). The intervention was associated with significant increases in limit setting at 2-months (estimate = 0.17, *SE* = 0.08, *p* = 0.02), controlling for baseline limit setting, although intervention assignment was not significantly related to depression (estimate = 0.03, *SE* = 0.07) or conduct problems (estimate = 0.01, *SE* = 0.20) at 4-months, controlling for baseline levels of each outcome. Depression at 4-months was significantly related to limit setting at 2-months (estimate =  − 0.13, *SE* = 0.06, *p* = 0.04), although the indirect effect of treatment on youth depression via limit setting was not significant (estimate =  − 0.02, *SE* = 0.01, *p* = 0.13). Conduct problems at 4-months were not significantly associated with improvements in limit setting at 2-months (estimate =  − 0.06, *SE* = 0.06), and the indirect effect of treatment via limit setting was also not significant (estimate =  − 0.03, *SE* = 0.04).

## Discussion

The current study evaluated the effects of an adaptation of the Family Check-Up Online (FCU-O) that aimed to support caregivers and children in coping with pandemic-related stressors. Building on a previous study (Connell & Stormshak, 2023) that demonstrated the adapted FCU-O had short-term intervention effects 2-months post-baseline on caregiver stress and parenting behaviors, the current study tested intervention effects at 4-months post-baseline on parenting behaviors and caregiver stress as well as on child outcomes, depressive symptoms and conduct problem behaviors. The current study also tested whether change in parenting behaviors and caregiver stress mediated intervention effects on child outcomes.

Supporting results from the 2-months post-test evaluation, the current study demonstrated FCU-O effects on intervention-targeted mediators at 4-months post-baseline. Specifically, the FCU-O reduced caregiver perceived stress and negative parenting, and FCU-O effects on proactive parenting were trending in a favorable direction, though they did not reach significance. While research on the in-person FCU supports direct effects on youth depression and conduct problems (Connell & Dishion, 2008; Shelleby et al., 2018), there were no direct intervention effects on any child outcomes in the current study. Differences between this study and prior in-person FCU studies on child outcomes may be attributable to differences between the in-person and online versions of the intervention. However, indirect effects in this study are consistent with our theoretical model and prior research specifying that the FCU improves parenting and family processes, which link to changes in child emotional and behavioral problems (Dishion et al., 2008; Fosco et al., 2014; Rudo-Stern et al., 2019). Although the FCU-O had no direct effects on child outcomes, we proceeded with tests of indirect effects to understand if our results supported the FCU theoretical model. Specifically, testing indirect effects offered an opportunity to assess whether our results supported the action theory (i.e., linking the FCU-O to program mediators) and conceptual theory (i.e., linking FCU-O targeted mediators to youth outcomes) underlying the FCU-O intervention model (O’Rourke & MacKinnon, 2018). Consistent with previous research, there was support for parenting and family processes (i.e., stress) as mechanisms of change in the FCU-O model. However, this is the first study to support caregiver stress as an FCU mechanism of change on child outcomes. The FCU-O offered skills to help caregivers manage their stress and skills to communicate with children about stress, anxiety, and depression, such as listening and problem-solving. Consistent with emotion socialization theory (Eisenberg et al., 1998), caregiver management of their stress may have been instrumental in helping youth regulate their emotions.

### A Virtual Randomized Control Trial (RCT) of a Telehealth Intervention: Lessons Learned

Most RCTs involving university-school partnerships to evaluate evidence-based interventions have relied on in-person interactions to build partnerships, recruit participants, collect data, or deliver the intervention. In-person interactions offer opportunities for trust-building, which promotes collaboration and is instrumental to research-community partnerships. However, the COVID-19 pandemic and corresponding physical distancing guidelines forced researchers to transition to conducting research virtually. This transition offered significant challenges, but there were also opportunities for innovation. For example, our research team coordinated efforts with a public health initiative (i.e., district-wide dissemination of COVID-19 testing kits) to reach caregivers and invite their participation in this study. Although this study was conducted entirely virtually, it is noteworthy that our retention from randomization to the 4-month follow-up was 76% for the control group and 84% for the intervention group, which is comparable to our in-person RCTs. Moreover, caregivers in the intervention condition completed more than five coaching sessions. Collectively, our results suggest that virtually conducted RCTs of virtually implemented interventions can be successful with respect to engagement and outcomes. It is worth noting that our research team had long-standing collaborative partnerships and established trust with many of the districts represented in this study, which may have contributed to our successful recruitment efforts (Metz et al., 2022).

### Clinical Implications and Future Directions

Parenting interventions are the most effective tool for improving youth outcomes, with research supporting effects on parenting and family relationships that link to long-term effects on externalizing and internalizing disorders among ethnically and socioeconomically diverse youth (Connell et al., 2021, 2022). Although in-person has been the primary modality for delivering parenting interventions, this modality requires substantial resources and is prone to poor rates of participation that diminish intervention effects, especially given current staffing shortages in community mental health settings. Telehealth models are effective in improving parenting and family functioning as well as child outcomes, and they have the capacity to reduce participation barriers and significantly increase intervention reach, particularly for underserved populations who may experience disproportionate barriers to accessing services (Spencer et al., 2020). This study’s results showing that caregivers high on SES-related risk engaged in the FCU-O more than other caregivers suggest a telehealth parenting intervention can increase service reach during periods of heightened stress for families who may be underserved.

Preventive interventions show differential effectiveness depending on caregiver, child, and family characteristics (van Aar et al., 2019). While previous research shows that the in-person FCU is most successful with low-income families experiencing high levels of risk (Pelham et al., 2017), there is no current research focused on understanding variables that explain variability in caregiver responsiveness to the FCU-O. Telehealth interventions minimize the impact of participation barriers, such as transportation and childcare, which are disproportionately experienced by high-risk families, perhaps resulting in higher levels of engagement in telehealth services among high-risk parents (Shelleby et al., 2018). However, there is limited research examining associations between contextual risk and engagement in telehealth interventions. Research comparing caregiver responsiveness to in-person versus telehealth interventions is even more limited. An understanding of which caregivers may be more responsive to parenting telehealth services and which may be more likely to engage with an in-person intervention has implications for increasing intervention reach for families that may benefit the most from parenting interventions.

In the current study, caregivers had access to a trained coach, who was a clinician and research team member. In a previous study (Stormshak et al., 2019), intervention effects were more robust for caregivers randomized to FCU-O plus coach versus FCU-O alone. However, evidence suggests that the effects of online parenting interventions with and without telehealth coaching do not differ (Spencer et al., 2020). Additional research examining FCU-O effects with and without coaching and experimentally manipulating coaching dosage would be valuable to understand the intervention intensity needed to attain effects for caregivers varying on contextual factors. In addition, research exploring the varying effectiveness of FCU-O coaches based on their differences in education and training would be important to understand the required credentials for FCU-O coaches. This information would have implications for FCU-O costs and dissemination.

### Limitations

This study has some noteworthy limitations. First, the sample is homogenous, with most caregivers identifying as White. Most caregivers are also college-educated, and the median family income is above poverty level. Also, most caregivers identified as female, limiting the generalization of results to male caregivers. One potential reason contributing to our oversampling of White and higher-income families is our recruitment strategy. Given that we invited study participation via a flyer included in COVID-19 testing kits, our recruitment strategy may have disproportionately reached White, higher-income caregivers who were more inclined to participate in the SARS-CoV-2 testing. There is some evidence that mistrust in healthcare systems and government was a barrier to SARS-CoV-2 testing among persons representing marginalized groups (Reitsma et al., 2021). Indeed, our sample had a greater proportion of White caregivers (84.6%) relative to students in participating districts (64.2%). However, it is notable that the representation of Latine caregivers in our sample (20.4%) was comparable to participating districts (20.9%). Given the sample’s homogeneity, analyses examining moderating effects of caregiver race, ethnicity, SES, and gender on intervention outcomes are limited. Future FCU-O studies should focus on engaging racially and ethnically diverse families to evaluate the FCU-O with more diverse populations. Future FCU-O studies that engage male caregivers are also needed. Another limitation is that outcomes were limited to caregiver self-report measures. This approach is consistent with the parent-centered intervention and the remote-delivery design, but the inclusion of independent informants including youth report on youth outcomes would enhance confidence in our results. In addition, the interim between baseline and the 4-month follow-up, when we measured child outcomes, may not have been long enough for intervention effects on child outcomes to emerge, which may be a potential reason we did not observe effects on child outcomes. Lastly, it is promising that FCU-O effects on mediators at the 2-month assessment were generally replicated at 4-months, but the follow-up intervention window is still short-term. We need research evaluating FCU-O’s long-term effects.

### Conclusions

This study highlights the effects of the FCU-O on parenting and family processes that link to improved child outcomes. Conducted during the pandemic, a time of unprecedented family stress, the results are significant as they suggest that online and telehealth supports, like the FCU-O, are effective and important public health interventions for families under extreme stress. In the current context, with youth mental health concerns at an all-time high and a significant decline in available mental health professionals, dissemination of effective, low-cost, and brief telehealth interventions is urgent. This study also indicates that fully virtual RCTs can be successful with respect to engagement and outcomes.

## Supplementary Information

Below is the link to the electronic supplementary material.

Supplementary file1 (DOC 220 KB)

Supplementary file2 (DOCX 26 KB)

Supplementary file3 (DOCX 388 KB)

## Data Availability

De-identified data supporting this study's findings are available from the corresponding author upon reasonable written request and contingent on a data transfer agreement.
