# Supplementary material for: Family Check-Up Online: Effects of a Virtual Randomized Trial on Parent Stress, Parenting, and Child Outcomes in Early Adolescence
Source: Prev Sci. 2024 Sep 24;27(1):119–30. doi: 10.1007/s11121-024-01725-3 (PMC12906586; doi:10.1007/s11121-024-01725-3)
Supplement: Supplementary file 2 — Supplementary file2 (DOCX 26 KB) [file 11121_2024_1725_MOESM2_ESM.docx]

**Supplemental Table 1**

*Sample Demographics by Intervention Assignment*

|  | Wait-list (n = 87) | |  | Intervention (n = 74) | |
| --- | --- | --- | --- | --- | --- |
|  | *n* | % |  | *n* | % |
| Parent’s gender (female) | 83 | 95.4 |  | 70 | 94.6 |
| Child’s gender (female) | 46 | 52.9 |  | 36 | 48.6 |
| Parent’s race/ethnicity |  |  |  |  |  |
| White/Caucasian | 74 | 85.1 |  | 62 | 83.7 |
| Black/African American | 0 | 0 |  | 1 | 1.4 |
| Asian | 2 | 2.3 |  | 1 | 1.4 |
| Multiracial | 9 | 10.3 |  | 10 | 13.5 |
| Unknown | 2 | 2.3 |  | 0 | 0 |
| Parent Hispanic/Latine | 18 | 20.7 |  | 14 | 18.9 |
| Parent’s education level |  |  |  |  |  |
| Less than high school degree | 3 | 3.4 |  | 2 | 2.8 |
| High school degree or GED | 7 | 8 |  | 6 | 8.1 |
| Partial college | 9 | 10.3 |  | 10 | 13.5 |
| 2-year associate’s degree | 6 | 6.9 |  | 10 | 13.5 |
| 4-year college degree | 33 | 37.9 |  | 24 | 32.4 |
| Graduate/professional training | 29 | 33.3 |  | 22 | 29.7 |
| Child’s grade |  |  |  |  |  |
| 4^th^ | 1 | 1.1 |  | 2 | 2.7 |
| 5^th^ | 6 | 6.9 |  | 3 | 4.1 |
| 6^th^ | 42 | 48.3 |  | 26 | 35.1 |
| 7^th^ | 20 | 23 |  | 26 | 35.1 |
| 8^th^ | 18 | 20.7 |  | 15 | 20.3 |
| 9^th^ | 0 | 0 |  | 2 | 2.7 |
| Primary caregiver single | 18 | 20.7 |  | 10 | 13.5 |
| Family below poverty-level | 13 | 14.9 |  | 11 | 14.9 |
| Primary caregiver unemployed | 6 | 6.9 |  | 5 | 6.8 |

**Supplemental Table 2**

*Parameter Estimates from Multilevel Models*

|  | **Perceived Stress** | **Negative Parenting** | **Proactive Parenting** | **Limit Setting** | **Youth Conduct Problems** | **Youth Depressive Symptoms** |
| --- | --- | --- | --- | --- | --- | --- |
| **Parameter** | *est (SE), p-value* | *est (SE), p-value* | *est (SE), p-value* | *est (SE), p-value* | *est (SE), p-value* | *est (SE), p-value* |
| Intercept | **1.11 (.48), p = .022** | **1.50 (.48), p = .002** | **4.54 (.50), p < .001** | **3.73 (.41), p < .001** | 2.74 (1.72), p = .11 | .64 (.45), p = .156 |
| SES Risk | **.11 (.03), p = .001** | **.13 (.03), p < .001** | .02 (.04), p = .68 | -.00 (.03), p = .938 | **.41 (.12), p < .001** | **.11 (.03), p = .001** |
| Racial/ethnic Minority | -.18 (.09), p = .054 | -.09 (.09), p = .304 | -.01 (.04), p = .945 | -.052 (.076), p = .677 | -.60 (.32), p = .063 | -.12 (.08), p = .149 |
| Age (months) | .003 (.003), p = .424 | -.01 (.003) p = .132 | **-.01 (.003), p = .003** | -.01 (.003), p = .065 | -.01 (.01), p = .538 | -.001 (.003), p = .662 |
| Group | **.21 (.09), p = .021** | .16 (.09), p = .07 | **-.31 (.096), p = .002** | **-.21 (.08), p = .012** | -.11 (.31), p = .715 | -.04 (.08), p = .647 |
| Time |  |  |  |  |  |  |
| * Baseline vs. 2 mo. | **.29 (.05), p = .001** | **.423 (.05), p < .001** | **-.14 (.07) p = .038** | -.118 (.063), p = .063 | **.40 (.15), p = .010** | **.19 (.05), p < .001** |
| * 2 mo. vs. 4 mo. | -.01 (.05), p = .797 | .08 (.05), p = .099 | -.01 (.07), p = .932 | .03 (.059), p = .644 | -.03 (.14), p = .848 | .05 (.05), p = .305 |
| Group x Time |  |  |  |  |  |  |
| * Control (Baseline vs. 2 mo.) | **-.22 (.07), p = .004** | **-.31 (.07), p < .001** | **.18 (.09), p = .053** | .12 (.09), p = .153 | -.074 (.21), p = .729 | -.05 (.07), p = .472 |
| * Control (2 mo. vs. 4 mo.) | .07 (.07), p = .342 | .01 (.07), p = .92 | .02 (.09) p = .850 | -.006 (.08), p = .941 | .21 (.20), p = .282 | .01 (.07), = .823 |

*Note:* Statistically significant values are bolded. A risk index related to SES was created based on six dichotomous indicators: high school diploma or GED, the parent is single, income below the poverty threshold (relative to family size), recipient of financial assistance (e.g., food stamps), currently unemployed, and the family’s home is overcrowded (bottom one-third of the sample for room-to-occupant ratio).

**Supplemental Table 3**

*Correlations Between Minutes Using FCU Online Support, Minutes in Virtual Coaching, and Socioeconomic Status (SES) Among Intervention Participants (N=74).*

|  | 1. | 2. | 3. |
| --- | --- | --- | --- |
| 1. Minutes using mobile application | -- |  |  |
| 1. Minutes spent on virtual coaching | .28* | -- |  |
| 1. SES Risk | .21^†^ | .24* | -- |

*Note.* A risk index related to SES was created based on six dichotomous indicators: high school diploma or GED, the parent is single, income below the poverty threshold (relative to family size), recipient of financial assistance (e.g., food stamps), currently unemployed, and the family’s home is overcrowded (bottom one-third of the sample for room-to-occupant ratio).

**p* < .05; ^†^*p* < .10

**Supplemental Table 4**

*Correlations for baseline variables*

|  | | 1. | 2. | 3. | 4. | 5. | 6. | 7. | 8. | 9. | 10. |
| --- | --- | --- | --- | --- | --- | --- | --- | --- | --- | --- | --- |
| 1. Intervention group | | -- |  |  |  |  |  |  |  |  |  |
| 1. Youth age (months) | | .08 | -- |  |  |  |  |  |  |  |  |
| 1. Youth gender (female) | | -.05 | -.12 | -- |  |  |  |  |  |  |  |
| 1. Racial/ethnic minority status | | -.02 | .06 | -.03 | -- |  |  |  |  |  |  |
| 1. SES Risk | | -.03 | -.02 | .05 | .36^**^ | -- |  |  |  |  |  |
| 1. Parent perceived stress | | .01 | .01 | .07 | -.03 | .16^*^ | -- |  |  |  |  |
| 1. Negative parenting | | .11 | -.14 | .11 | .01 | .20^**^ | .43^**^ | -- |  |  |  |
| 1. Proactive parenting | | .09 | -.18^*^ | -.04 | .04 | .16^*^ | -.01 | -.04 | -- |  |  |
| 1. Limit-setting | | .08 | -.10 | .00 | -.08 | .07 | -.19^*^ | -.20^*^ | .54^**^ | -- |  |
| 1. Youth conduct problems | | .04 | -.08 | .12 | -.03 | .24^**^ | .24^**^ | .55^**^ | .04 | .01 | -- |
| 1. Youth depression | | .08 | -.03 | -.06 | -.02 | .25^**^ | .30^**^ | .33^**^ | .30^**^ | .07 | .42^**^ |
|  | * *p* < .05; ** *p* < .01 | | | | | | | | | | |
