# Supplementary material for: Family Check-Up Online: Effects of a Virtual Randomized Trial on Parent Stress, Parenting, and Child Outcomes in Early Adolescence
Source: Prev Sci. 2024 Sep 24;27(1):119–30. doi: 10.1007/s11121-024-01725-3 (PMC12906586; doi:10.1007/s11121-024-01725-3)

**Supplemental Figure 1**

*A Visual Representation of The Family-Check-Up Online COVID-19 Pandemic Adaptation*

| When caregivers log on to the middle school version of the Family Check-Up Online, they will first be directed to an introductory video, “How to Use the Family Check-Up”, that highlights how the program works and what caregivers can expect as they navigate the program. |  | 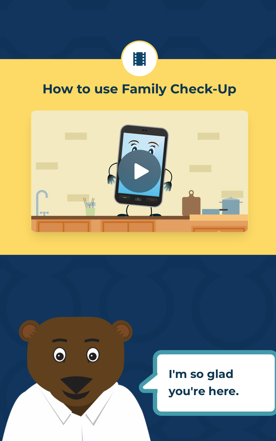 |
| --- | --- | --- |
|  |  |  |
| After the introductory video, caregivers are directed to the 5 program modules: Healthy Behaviors, Positive Parenting, Rules and Consequences, Support for School Success, and Communication. |  | 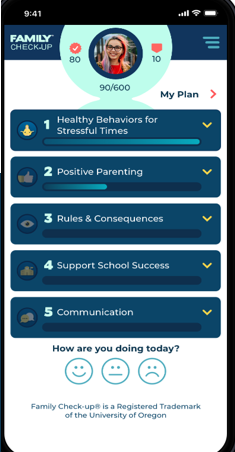 |
|  |  |  |
| Each module addresses a number of content areas and skills and takes about 15-20 minutes to navigate. For example, the picture to the right illustrates the content areas covered in the Healthy Behaviors for Stressful Times module. |  | 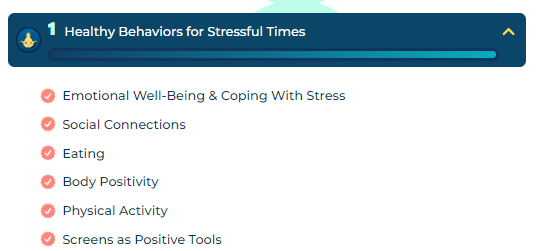 |
|  |  |  |
| Caregivers will begin each module by taking a short survey. An example item from the Healthy Behaviors for Stressful Times module is presented here. |  | 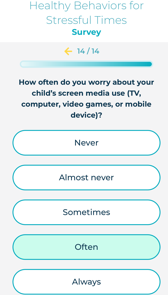 |
|  |  |  |
| A graphic that summarizes caregiver perceptions of strengths and challenge areas based on survey responses is auto generated. The picture to right illustrates an example of the graphic feedback for the Healthy Behaviors for Stressful Times module. In the example to the right, “physical activity” is a strength for this caregiver, and “focus areas” are “body positivity” and “screens as positive tools”. The other areas, “emotional well-being”, “coping with stress”, “social connections”, and “eating” are areas that need attention, meaning that the caregiver is encouraged to attend to these areas proactively to make positive changes for their child and family now to prevent these areas from becoming focus or problem areas in the future. The caregiver can clink on the icon- 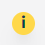- to learn more about the categories “focus area”, “needs attention”, and “area of strength”. When caregivers slect “Done”, they are taken to the module content. |  | 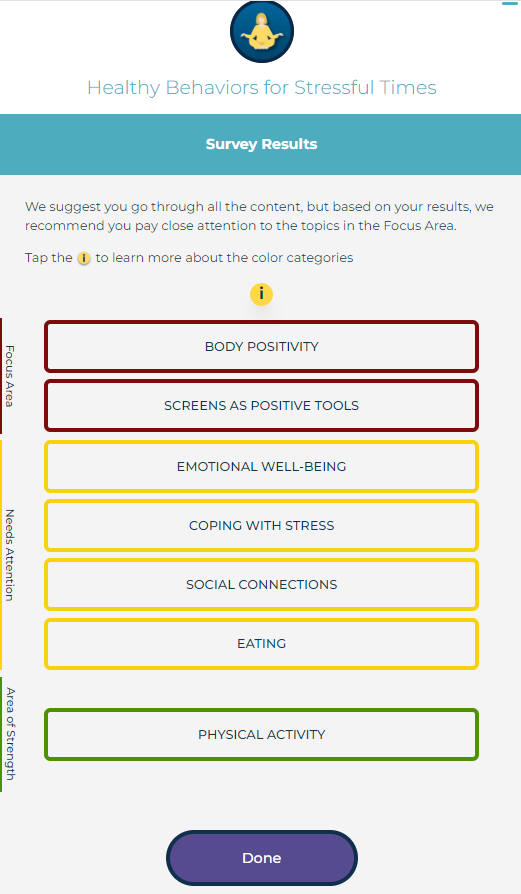 |
|  |  |  |
| The module begins with didactic content about skills addressed in the module. |  | 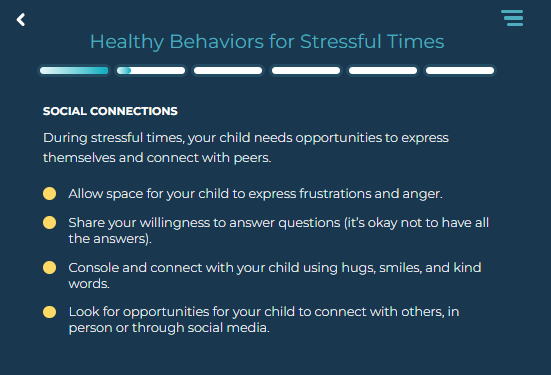  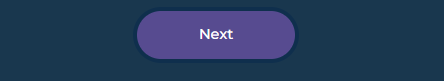 |
| Followed by a video model of an animated character demonstrating targeted skills. |  | 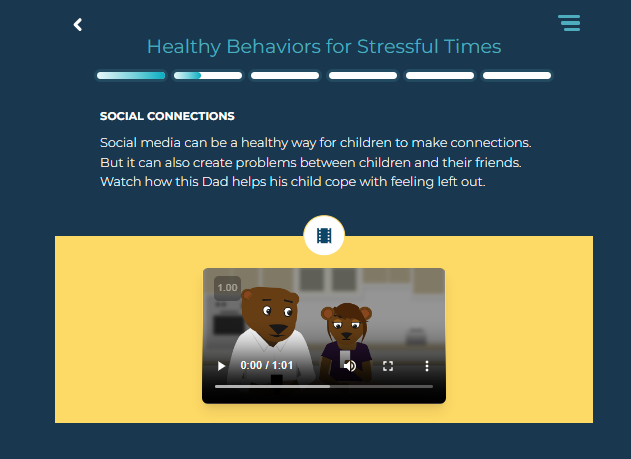  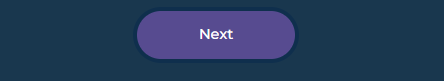 |
| Then, the caregiver has interactive practice opportunities to encourage caregiver skills practice. Caregivers can stop mid-module and save their progress. |  | 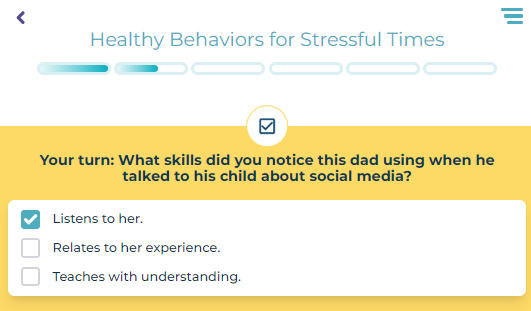  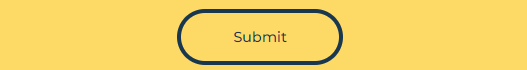 |

**Supplemental Figure 2**

*Mediation Model*


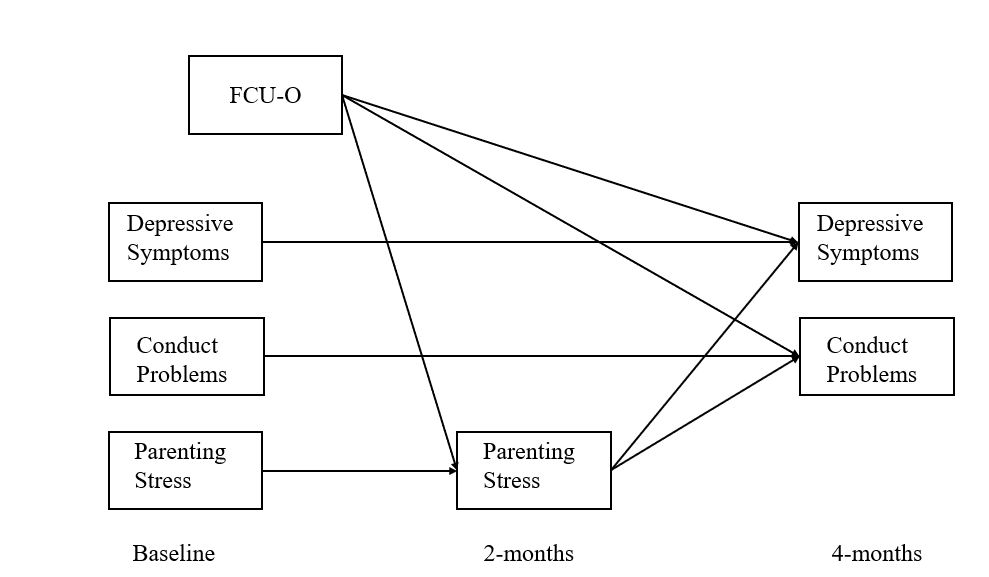

Supplement: Supplementary file 3 — Supplementary file3 (DOCX 388 KB) [file 11121_2024_1725_MOESM3_ESM.docx]
